# The Role of Brain Activity in Characterizing Successful Reading Intervention in Children With Dyslexia

**DOI:** 10.3389/fnins.2022.898661

**Published:** 2022-06-13

**Authors:** Anthony J. Krafnick, Eileen M. Napoliello, D. Lynn Flowers, Guinevere F. Eden

**Affiliations:** Center for the Study of Learning, Department of Pediatrics, Georgetown University Medical Center, Washington, DC, United States

**Keywords:** dyslexia, reading disability, intervention, fMRI, children

## Abstract

Studies of reading intervention in dyslexia have shown changes in performance and in brain function. However, there is little consistency in the location of brain regions associated with successful reading gains in children, most likely due to variability/limitations in methodologies (study design, participant criteria, and neuroimaging procedures). Ultimately for the results to be meaningful, the intervention has to be successful, be assessed against a control, use rigorous statistics, and take biological variables (sex) into consideration. Using a randomized, crossover design, 31 children with dyslexia were assigned to a phonological- and orthographic-based tutoring period as well as a within-subjects control period to examine: (1) intervention-induced changes in behavior (reading performance) and in brain activity (during reading); and (2) behavioral and brain activity pre-intervention data that predicted intervention-induced gains in reading performance. We found gains in reading ability following the intervention, but not following the control period, with no effect of participants’ sex. However, there were no changes in brain activity following the intervention (regardless of sex), suggesting that individual brain changes are too variable to be captured at the group level. Reading gains were not predicted by pre-intervention behavioral data, but were predicted by pre-intervention brain activity in bilateral supramarginal/angular gyri. Notably, some of this prediction was only found in females. Our results highlight the limitations of brain imaging in detecting the neural correlates of reading intervention in this age group, while providing further evidence for its utility in assessing eventual success of intervention, especially if sex is taken into consideration.

## Introduction

Developmental dyslexia is a common learning disability, affecting approximately between 5 and 13% of the general United States population (Katusic et al., 2001). It is defined by difficulties in word recognition and word decoding, that are incongruent with other cognitive skills, classroom experience, and motivation to learn how to read (Lyon et al., 2003). The word decoding problems (sounding out of novel words) are believed to be due to difficulties with understanding how sounds in speech are isolated, manipulated, and recalled (phonological coding or phonological awareness; Scarborough and Brady, 2002) and other difficulties with representing the speech stream (Peterson and Pennington, 2012), therefore impeding their mapping onto their corresponding graphemes during reading. Further, word form recognition is also impaired in dyslexia, not only as a consequence of poor phonological coding skills, but also because of difficulties in establishing a “sight word vocabulary” through memorization of the visual (orthographic) word forms (Badian, 1995, 2001). Brain imaging studies have revealed hypoactivation in brain regions associated with phonological mapping in temporo-parietal cortex (TPC) and in regions associated with visual word form recognition in the occipito-temporal cortex (OTC) (Pugh et al., 2001; Sandak et al., 2004; Eden et al., 2015). The role of these areas in reading acquisition and in dyslexia continue to be an active area of discussion (Richlan, 2012).

When it comes to addressing the reading difficulties of children with dyslexia, explicit instructions in phonological coding (Alexander and Slinger-Constant, 2004), letter-speech sound training (Brem et al., 2010; Kyle et al., 2013) and orthographic facilitation (Baron et al., 2018) are often key elements in the interventions administered. Ideally, such tutoring occurs in small groups (or one-on-one) with an emphasis on early intervention (Wanzek and Vaughn, 2007). Understanding the kinds of reading interventions that work and how they work, is of continued interest. Investigations into the neural correlates of reading intervention in dyslexia have shown widespread increases in brain activity in children (Aylward et al., 2003; Temple et al., 2003; Shaywitz et al., 2004; Richards et al., 2006; Meyler et al., 2008; Odegard et al., 2008; Gebauer et al., 2012) and adults (Eden et al., 2004). A narrative review by Barquero et al. (2014) describes these to include left and right hemisphere inferior, middle, and superior frontal gyri, superior and middle temporal gyri, occipital cortices, inferior parietal lobule, post central gyrus, and insulae (Barquero et al., 2014). Notably it has been suggested that gains in reading in dyslexia are associated with increases in left-hemisphere regions typically involved in reading, while other regions, such as right frontal cortex (Temple et al., 2003; Richards et al., 2006; Meyler et al., 2008), perhaps serve in a compensatory role (Shaywitz et al., 2004; Hoeft et al., 2007; Barquero et al., 2014). However, a meta-analysis of intervention neuroimaging research demonstrates a lack of convergence across studies for many of these implicated brain regions (Barquero et al., 2014). The strongest results from this meta-analysis of eight studies (173 participants) were left thalamus (three studies contributing), right insula/inferior frontal gyrus (four studies contributing), and left inferior frontal gyrus (three studies contributing). Right posterior cingulate and left middle occipital gyrus were also identified (though with only two contributing studies). Notably, other left-hemisphere regions typically involved in reading, that is left OTC and PTC, were not found to change. A more recent meta-analysis of changes in brain activation following reading intervention of eight studies (151 participants) and differing from the Barquero meta-analysis by two out of eight studies, found no results (Perdue et al., 2022).

There are also methodological limitations that question the validity of prior findings on changes in brain activity following reading intervention in dyslexia. As a whole, the imaging thresholds used are far less stringent than those used today. Of the studies in children and adolescents included in the Barquero and Perdue meta-analyses, only four used any correction of cluster size in their whole brain analysis (Aylward et al., 2003; Gebauer et al., 2012; Heim et al., 2015; Partanen et al., 2019), and voxel level thresholds vary considerably. Another concern is the variability in reported behavioral gains associated with the interventions. Not all studies report on changes in single word reading ability (though most report comprehension level data), and only two have examined whether these gains persist in the long-term (Shaywitz et al., 2004; Meyler et al., 2008). Importantly, while many studies of dyslexia include a control group, none include a within-subject control intervention to assess specificity of these changes, a design that is considered best practice in clinical research.

In addition to examining the brain bases for reading disability and successful reading intervention, neuroimaging has also been used in a small number of studies to examine whether brain function can be used to “predict” later reading outcome in typically reading children as well as children with dyslexia; and these studies have been done either with or without conducting a formal intervention. For example, in typically developing children both left visual word system (fusiform gyrus) event-related potentials and functional magnetic resonance imaging (fMRI) signal attained during a lexical decision task in kindergarten (prior to learning to read) were found to be predictive of how well children (who participated in a speech-sound association training) could read in second grade (Bach et al., 2013). Specific to dyslexia, a study in children and adolescents with dyslexia found that right inferior frontal gyrus activation during a written word rhyming task predicted single word reading measures 2.5 years later (no intervention was provided) (Hoeft et al., 2011). Two studies have examined reading intervention in children with dyslexia and tested whether gains in reading following the intervention were predicted by pre-intervention brain activity. One study found gains in untimed pseudoword reading were predicted by pre-intervention activity during a phonological processing task in left inferior frontal gyrus, and gains in timed word reading were predicted by activity in left and right inferior frontal gyri (Farris et al., 2016). Another study found gains in basic reading were predicted by pre-intervention functional connectivity between middle temporal gyrus and left inferior parietal lobule during a lexical decision task (Aboud et al., 2018). Like studies investigating changes in brain activity with intervention, the use of cluster-level correction for the whole-brain analysis is mixed, with only the last two of the above mentioned studies using cluster level correction (Farris et al., 2016; Aboud et al., 2018).

There have been recent calls to pay more attention to sex as a biological variable in all research (Cahill, 2006, 2012) and especially in research of language processing and dyslexia (Ramus et al., 2018; Krafnick and Evans, 2019). Sex has played a role in language research, where converging evidence suggest sex-specific differences in language acquisition and development (Martin and Hoover, 1987; Bornstein et al., 2000; Dionne et al., 2003), as well as sex-specific organization of the brain for language (Shaywitz et al., 1995; Jaeger et al., 1998; Kansaku and Kitazawa, 2001; Burman et al., 2008). For example, males have been shown to have more left-lateralized activation of perisylvian brain regions during language tasks, whereas females activate bilateral perisylvian brain regions (Shaywitz et al., 1995; Jaeger et al., 1998).

Specific to dyslexia, prevalence differs amongst boys and girls with odds ratios ranging from 1.39 to 3.19 in favor of higher prevalence in boys (Rutter et al., 2004; Quinn and Wagner, 2015), even when controlling for ascertainment bias (Liederman et al., 2005; Quinn and Wagner, 2015). Neuroimaging studies of dyslexia have on average recruited more male subjects, as reflected in 65% male participants contributing to the meta-analysis in children, and 95% to the meta-analysis in adults reported by Richlan et al. (2011), and 59% of subjects in a meta-analysis of neuroimaging studies of reading intervention (Barquero et al., 2014). Most importantly, evidence of sex-specific differences in dyslexia from studies of gray matter volume (Evans et al., 2014) and cortical thickness (Altarelli et al., 2013; Clark et al., 2014) suggest that the brain bases of dyslexia may not be the same in males and females, with females, but not males, showing differences in anatomy in brain regions associated with early sensory processing (Altarelli et al., 2013; Clark et al., 2014; Evans et al., 2014). However, sex has not been accounted for in investigations into the brain-based correlates of successful reading intervention in dyslexia. Sex-specific differences in dyslexia prior to an intervention could lead to sex-specific differences in the neural correlates of successful intervention. Critical to this study, failure to identify any antagonist interactions for sex could result in failure to register significant (sex-specific) changes following intervention. That is, if sex-specific changes are in opposite directions, the changes during intervention could appear small or non-existent. Lastly, if the behavioral response to reading intervention is the same for males and females, it does not mean that the neural substrates underlying that change in performance is the same for both sexes (Cahill, 2006).

In the present study we report behavioral data for reading and reading-related skills as well as fMRI data during a word processing task in 31 children with dyslexia. These data were acquired in all children prior to and following (i) an intensive intervention focused on promoting reading through phonological and orthographic skills, and (ii) an intensive intervention focused on promoting math (active control) or, instead, a null period (developmental control), using a randomized, crossover design. Our study of dyslexia allowed us to ask: (1) What are the brain activation changes that follow a successful reading intervention, and are these changes specific to the reading intervention? (2) Can brain activity during reading indicate whether children will subsequently reap benefits from the reading intervention? And (3) Are these findings affected by sex? Together these findings should advance our understanding of the location and specificity of the neural correlates that underlie successful reading intervention in males and females with dyslexia, as well as whether brain activity signals a readiness to benefit from such a reading intervention.

## Materials and Methods

### Participants

Thirty-one dyslexic children (14 female; age average 9.6 and range 7.4–12.6 years) were recruited from a private school specializing in students with learning disabilities. School records were used to identify children who had a score of less than or equal to 92 on the Woodcock–Johnson Test of Achievement III Letter-Word Identification (W-J WID) and/or Word Attack (W-J WA) (Woodcock et al., 2001), and a documented diagnosis of dyslexia. In order to be included in the study, children had to score at least 80 on Verbal, Performance, and Full IQ on the Wechsler Abbreviated Scale of Intelligence (WASI) (Wechsler, 1999). All children were in good health and free of other developmental disabilities, neurological and psychiatric disorders or any disease affecting brain function, except for ADHD (children taking medication for ADHD had to refrain taking it prior to the scans). Other exclusion criteria included contraindications to MRI scanning such as metallic implants or severe claustrophobia. fMRI data for some of these children using the same reading task have been published previously in a comparison with typically reading children (Olulade et al., 2015).

### Behavioral Tests

All subjects received a battery of psychoeducational tests to evaluate intelligence quotient (IQ), reading, and skills that are related to reading. Except for IQ, the entire testing battery was administered at all three visits (prior to and following interventions). The WASI (Wechsler, 1999) was used to measure IQ. The Woodcock–Johnson Test of Achievement III was used to assess reading ability: Word Identification (W-J WID) subtest for single real word reading, Word Attack (W-J WA) subtest for single pseudoword reading, and Passage Comprehension (W-J PC) for understanding of written text (Woodcock et al., 2001). In addition, we measured skills that play a role in acquiring reading and are typically impaired in dyslexia: the Lindamood Auditory Conceptualization Test (LAC) for phonemic awareness (Lindamood and Lindamood, 1971), the Rapid Automatized Naming test (RAN L&N and C&O) for naming fluency of letters/numbers and colors/objects (Denckla and Rudel, 1976a,b), the Digit Span test for working memory (Wechsler, 1999), and the Symbol Imagery (SI) test for visual imagery (memory for letters and orthographic patterns) (Bell, 1997). These measures were used to gauge improvement in reading and reading-related skills, which were expected to increase following the reading intervention but not following the math intervention. To also assess changes in mathematical performance, we used the Calculation subtest for computational ability, the Math Fluency subtest for timed arithmetic, and the Applied Problems subtest of the Woodcock–Johnson Test of Achievement III (Woodcock et al., 2001) for mathematical word problems. All scores reported are standard scores (Population Mean = 100, SD = 15). Researchers acquiring these data were blind to each child’s group assignment.

### Study Design

**FIGURE 1:**
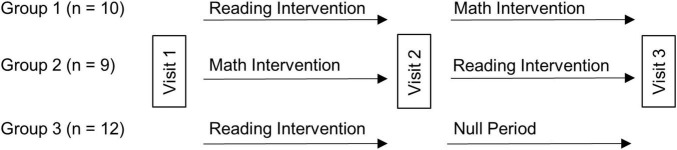
Intervention study design. Thirty-one children with dyslexia were randomly assigned to one of three intervention arms. All groups received the reading intervention, and either a math intervention (active control, Groups 1 and 2) or null period (developmental control, Group 3). Each visit consisted of acquisition of behavioral and imaging data, with 6-week intervention periods between Visits 1 and 2, and Visits 2 and 3.

The children were randomly assigned to one of three groups. Each of the three groups received the same reading intervention (3 h a day, for a total of 90 h). For Group 1 (*n* = 10) this 6-week reading intervention was followed by a 6-week math intervention, and for Group 2 (*n* = 9) it was preceded by a math intervention (math intervention was also 3 h a day, for a total of 90 h). As such, these 19 children received the intervention of interest (reading) and an active control intervention (math), with the order counterbalanced (a 10th child originally assigned to Group 2 left the study after it had begun). Group 3 (*n* = 12) received the same reading intervention followed by a 6-week null period (no intervention) to provide a developmental control period (Krafnick et al., 2011). As such, we would be able to weigh any benefits resulting from the reading intervention against the possibility of a Hawthorn effect and/or a placebo effect (by comparison to the active control math intervention). The latter effects could result from participating in a study that involves intensive work on the part of the participants as well as strong encouragement by others for their efforts. Further, both the reading intervention and the active control math intervention could be assessed relative to no intervention (null period) to be able to assess changes relative to the normal developmental changes that would occur during this time span. Three behavioral testing/scanning sessions were scheduled eight weeks apart (one prior to any intervention/control period and another after each intervention/control period; see Figure 1). One-way ANOVAs showed that randomization to group was successful in keeping the groups similar in age, IQ, reading, and reading-related skills prior to intervention (Table 1). As such, age and IQ were not included in the analyses looking at gains in performance measures following the interventions. Further, a Chi-square test revealed no significant difference in sex amongst the groups (Table 1). Most subjects (26 of the original 31) returned for behavioral testing 1 year later, allowing us to gauge long-term outcome of the intervention.

**TABLE 1:** Behavioral profile prior to intervention (Visit 1) for entire group and by intervention group.

	Mean (SD)					
	All subjects (*n* = 31)	Group 1 (*n* = 10)	Group 2 (*n* = 9)	Group 3 (*n* = 12)	*F*-statistic/Chi-square	*p*-Value
Age (years)	9.6 (1.5)	10.0 (1.6)	9.9 (1.5)	9.0 (1.3)	1.414	0.260
Sex (M/F)	14/17	7/3	4/5	3/9	4.46	0.107
IQ: Wechsler Abbreviated Scale of Intelligence (WASI)*					*F* (2,27)	
Verbal IQ	110.2 (9.0)	111.9 (11.6)	107.5 (11.4)	110.7 (3.4)	0.538	0.590
Performance IQ	101.9 (10.2)	100.7 (10.8)	104.8 (8.9)	101.1 (10.9)	0.403	0.672
Full IQ	106.9 (8.4)	107.2 (11.6)	106.8 (7.6)	106.8 (6.2)	0.007	0.993
**Measures of reading: Woodcock–Johnson**					*F* (2,28)	
Word Identification (single real words)	77.4 (8.0)	80.3 (7.9)	75.8 (7.3)	76.2 (8.6)	0.982	0.387
Word Attack (single pseudowords)	91.8 (6.4)	93.0 (5.1)	89.2 (6.0)	92.7 (7.5)	1.018	0.374
Passage Comprehension (reading comprehension)	78.4 (13.9)	84.4 (9.9)	77.0 (14.7)	74.4 (15.4)	1.528	0.235
**Measures of skills that support reading**					*F* (2,28)	
Lindamood Auditory Conceptualization Test (phonemic awareness)	98.4 (8.4)	102.4 (9.9)	96.1 (8.4)	96.8 (6.4)	1.783	0.187
Rapid Naming (naming fluency for letters and numbers)	78.6 (12.4)	84.4 (11.6)	77.8 (9.7)	74.3 (13.7)	1.974	0.158
Rapid Naming (naming fluency for colors and objects)	84.5 (12.4)	90.7 (13.5)	79.2 (10.1)	83.3 (11.7)	2.313	0.118
Digit Span (working memory)	93.7 (11.8)	99.0 (12.9)	92.8 (12.8)	90.0 (9.3)	1.693	0.202
Symbol Imagery (visual imagery/orthographic processing)	80.3 (9.7)	84.4 (9.8)	74.7 (11.8)	80.8 (8.5)	2.502	0.100

*WASI scores were missing for one subject.

### Reading and Math Interventions

All 31 children received the reading intervention Seeing Stars^®^ (Bell, 1997) purchased by us from Lindamood-Bell Learning Processes^®^ and delivered by their employees at the children’s school in small groups. This intervention uses a “multisensory approach” to promote integration of internal visual and phonological representations of letters and letter strings. The imagery portion increases in difficulty starting with single letter imagery and increasing through two and three syllable words. A tactile/motor portion involves finger tracing of visualized letters, and a language production portion involves aloud verbalization of letter and syllable sounds while they are finger traced in the air. The use of imagery/visualization in this reading intervention is based on several studies involving the use of imagery in reading, including self-report of imagery during reading (Long et al., 1989), imagery in semantic retrieval (Kosslyn, 1976) and the use of imagery to improve processing and comprehension (Linden and Wittrock, 1981; Sadoski, 1983).

The math intervention was On Cloud Nine^®^ (Bell and Tuley, 1997) and was delivered by the same Lindamood-Bell Learning Processes^®^ staff at the school. It utilizes a multisensory approach focusing on imagery, tracing and verbalization, similar to the reading intervention, but with a focus on numbers and number lines instead of letters and syllables, thus serving as a suitable active control for the reading intervention.

### Behavioral Analyses

To test for performance changes brought about by the interventions, we conducted 2 × 2 repeated measures ANOVAs with Time Point (pre- versus post intervention) as a within-subjects factor and sex (male versus female) as a between-subjects factor (Time Point × Sex). Each analysis was specific to a reading or reading-related measure and a particular intervention, e.g., children’s pre- and post-reading intervention data for a given measure were included as “Time Points” to examine changes during the reading intervention. We refer to Time Points here as opposed to Visits in the description of the intervention design above (Figure 1) because of the counterbalanced design. For example, some participants’ pre-reading intervention visit was Visit 1, whereas others it was Visit 2; to investigate changes following the reading intervention, we use data from each subject’s pre- and post-reading intervention Time Points. We employed a Holm–Bonferroni correction for multiple comparisons (Holm, 1979). These pre- versus post intervention comparisons are similar to those presented in previous reading intervention studies (that did not include a control period) and are presented here for the purpose of comparison with behavioral gains following intervention in those studies.

However, to test whether any such gains in reading during the reading intervention are significantly greater than any gains during the control period, we conducted a 2 × 2 repeated measures ANOVA using intervention (reading intervention period versus math intervention/null period) and performance measure (change in reading ability on W-J WID versus change in math ability on Calculation standard score) as within-subjects factors and tested for an interaction.

To test if performance measures predicted reading gains, the three reading and the five reading-related measures at Visit 1 (prior to any intervention), age, IQ, and sex were entered into a single multiple regression with change in reading ability on W-J WID standard score as the dependent variable.

Behavioral analyses and visualization were carried out in SPSS (IBM SPSS Statistics 22), jamovi (version 2.5.5), and Microsoft Excel.

### Functional Magnetic Resonance Imaging Data Acquisition and Preprocessing

During acquisition of fMRI data, subjects performed an implicit reading task (Price et al., 1996). The children saw single real words (Word) or false font strings (False Font) and responded with a button press in their right hand if the Word or False Fonts contained a “tall” letter or character (e.g., “alarm” contains the tall letter “l”) and a button press in their left hand if it did not (e.g., sauce has no tall letters). This task has been used previously in our studies of reading and reading disability (Turkeltaub et al., 2003, 2004; Olulade et al., 2013, 2015; Evans et al., 2016). Blocks of Word and blocks of False Font stimuli alternated (twice each) and were separated by blocks of Fixation. Blocks of Words and False Font contained 10 trials each, lasting 42 s, and Fixation blocks lasted 18 s each (with additional Fixation scans at the beginning and end of the run, resulting in a total scan time of four minutes, twenty-seven seconds). Each child underwent two scan acquisitions (two runs yielding 28 whole-head echo planar imaging (EPI) volumes for each condition, Word, False Font and Fixation) at three different times over the study (one at each visit as described above, see Figure 1).

Functional magnetic resonance imaging fMRI data was acquired using an EPI sequence using a 3 Tesla Siemens Trio whole-body MRI system [TE = 30 ms, TR = 3 s, 64 × 64 matrix, 192 mm FOV, 50 axial slices, 2.8 mm slice thickness (0.2 mm interslice gap) yielding 3 mm cubic voxels, flip angle 90°]. A high resolution, 3D T1-weighted MPRAGE image obtained at the outset of the study (Visit 1, prior to any intervention) on the same Siemens Trio whole-body MRI system was used to aid in anatomical localization of the fMRI data.

Pre-processing for functional analysis began by segmenting the subjects’ MPRAGE images and normalizing to a standard template brain (Montreal Neurological Institute, MNI). For all functional runs, the first five scans were removed, and the remaining scans were corrected for head motion by realigning to the mean image, co-registered to the subjects MPRAGE, normalized using the same parameters for the MPRAGE image and finally smoothed using a 6 mm × 6 mm × 5.8 mm Gaussian kernel. For each subject’s first level analysis, both runs were included, and contrasts were generated for the Word versus False Font condition, Word versus Fixation condition and False Font versus Fixation condition. Motion parameters and global mean signal were included as regressors of no interest to account for subject movement and global signal variation during each run.

### Functional Magnetic Resonance Imaging Group Level Analyses

All analyses were carried out in SPM (Statistical Parametric Mapping, Wellcome Trust Centre for Neuroimaging, London, United Kingdom). All group analyses (differences in activation pre- versus post the intervention, and activation to predict intervention-induced changes in reading performance) were performed on Words > False Font contrasts at an uncorrected height threshold of *p* < 0.001, and an extent threshold of *p* < 0.05 family wise error (FWE) corrected. For the analysis on activation to predict intervention-induced reading gains, the MarsBaR toolbox (Brett et al., 2002) was used to extract the signal from clusters identified in the analyses (described below), to display the mean percent signal change. Again, sex was included as a between-subjects factor in the intervention Time Point comparisons (same as in the behavioral analyses).

## Results

### Behavioral Measures Change Following the Reading Intervention

**FIGURE 2:**
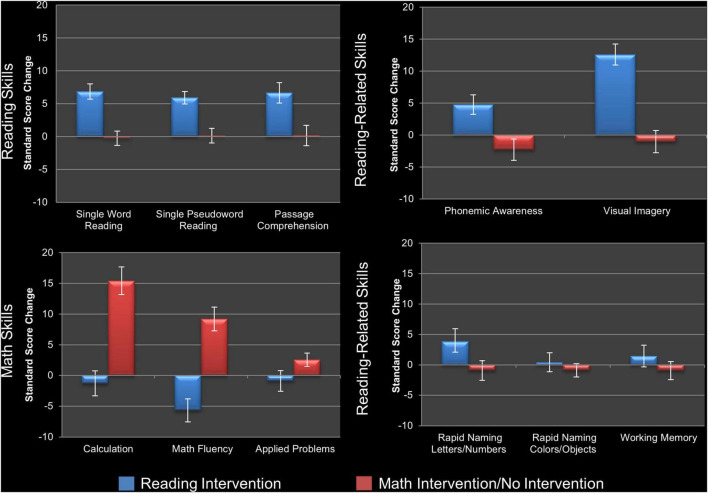
Changes in behavioral measures following intervention. Visualization of standard score changes in measures of reading skills (top left), phonological and orthographic reading-related skills (top right), other reading-related skills (bottom right), and math skills (bottom left) for the analyses reported in Table 2. Reading skills: single real word reading (Word Identification), single pseudoword reading (Word Attack), and reading comprehension (Passage Comprehension). Phonological and orthographic reading-related skills: phonemic awareness (Lindamood Auditory Conceptualization Test), and visual imagery/orthographic processing (Symbol Imagery). Other reading-related skills: naming fluency of letters/numbers and colors/objects (Rapid Automatized Naming test, and working memory (Digit Span). Math skills: computational ability (Calculation), timed arithmetic (Math Fluency), and mathematical word problems (Applied Problems). Error bars show standard error for the average change in standard score. For statistical tests see text.

To evaluate the impact of the reading intervention, the 2 × 2 repeated measures ANOVAs conducted on the standard scores of the three measures of reading and the five measures of reading-related skills, as well as the three math skills, immediately prior to and following the reading intervention (Time Point as within-subjects factor and Sex as between-subjects factor) found six of the eight reading/reading-related measures showed a significant main effect of Time Point and increased scores following the reading intervention (Table 2): real word reading (W-J WID), pseudoword reading (W-J WA), reading comprehension (W-J PC), phonemic awareness (LAC), naming fluency of letters/numbers (RAN L&N), and visual imagery (SI). One of the math measures (W-J Math Fluency) showed a significant main effect of Time Point, decreasing after the reading intervention. After Holm–Bonferroni correction (Holm, 1979), all three reading measures (real word reading, pseudoword reading, and passage comprehension), two of the five reading-related measures (phonemic awareness and visual imagery), and none of the math measures remained significant. As such the children made gains on a range of measures of reading, as well as the skills targeted by the intervention and known to promote reading acquisition (see Figure 2). There were no significant interactions for Time-Point × Sex.

**TABLE 2:** Changes in behavior following intervention.

	Mean (SD)		Main effect of time point	Time point × sex interaction
Pre versus post period of reading intervention	Pre	Post	*p*-Value	*p*-Value
***Measures of reading: Woodcock–Johnson***				
Word Identification (single real words)	77.5 (7.9)	84.4 (9.2)	*3.0 × 10^–6^	0.154
Word Attack (single pseudowords)	91.0 (7.0)	96.9 (7.3)	*1.0 × 10^–6^	0.610
Passage Comprehension (reading comprehension)	79.0 (12.3)	85.6 (7.8)	*2.3 × 10^–4^	0.710
***Measures of skills that support reading***				
Lindamood Auditory Conceptualization Test (phonemic awareness)	98.0 (8.4)	102.7 (10.7)	*0.005	0.123
Rapid Naming (naming fluency for letters and numbers)	79.0 (12.6)	82.7 (13.5)	0.029	0.399
Rapid Naming (naming fluency for colors and objects)	85.3 (12.6)	85.7 (15.6)	0.909	0.314
Digit Span (working memory)	92.3 (10.6)	93.7 (10.5)	0.533	0.879
Symbol Imagery (visual imagery/orthographic processing)	81.4 (8.4)	94.1 (12.3)	*1.0 × 10^–8^	0.077
***Math skills***				
Calculation (computational ability)	96.6 (13.5)	95.3 (10.6)	0.753	0.258
Math Fluency (timed arithmetic)	86.4 (12.8)	80.7 (15.0)	0.019	0.929
Applied Problems (mathematical word problems)	97.7 (9.5)	96.8 (7.9)	0.719	0.470

**Pre versus post period of math intervention/no intervention**	**Mean (SD)**			
	**Pre-MI/NI**	**Post-MI/NI**	***p*-Value**	***p*-Value**

***Measures of reading: Woodcock–Johnson***				
Word Identification (single real words)	82.9 (9.6)	82.6 (11.6)	0.924	0.146
Word Attack (single pseudowords)	96.0 (7.2)	96.1 (8.9)	0.923	0.874
Passage Comprehension (reading comprehension)	84.0 (10.7)	84.2 (10.6)	0.772	0.066
***Measures of skills that support reading***				
Lindamood Auditory Conceptualization Test (phonemic awareness)	102.4 (11.0)	100.1 (9.4)	0.226	0.088
Rapid Naming (naming fluency for letters and numbers)	83.0 (13.3)	82.1 (12.3)	0.392	0.515
Rapid Naming (naming fluency for colors and objects)	86.3 (15.1)	85.5 (13.9)	0.647	0.192
Digit Span (working memory)	93.7 (10.6)	92.7 (12.0)	0.725	0.408
Symbol Imagery (visual imagery/orthographic processing)	90.7 (14.6)	89.4 (12.8)	0.532	0.387
***Math skills^***				
Calculation (computational ability)	90.7 (12.0)	106.1 (15.3)	*3.0 × 10^–6^	0.445
Math Fluency (timed arithmetic)	80.8 (13.9)	90.0 (16.0)	*1.8 × 10^–4^	0.478
Applied Problems (mathematical word problems)	96.4 (7.2)	99.0 (8.6)	0.027	0.485

*p-Values in survive Holm–Bonferroni correction for multiple comparisons. ^Results limited to the two groups that received the math intervention, to evaluate effectiveness of the math intervention.

When the same analyses were conducted following the control periods (math and no intervention) none of the reading or reading-related measures changed (Table 2), demonstrating the specificity of the above-described effects of the reading intervention. To ensure this result was not because one type of control period (e.g., math intervention) had effects which were canceled out or diluted by opposite effects of the other control period (e.g., no intervention developmental control), a one-way ANOVA for Group on changes following the reading intervention was conducted for these two specific arms of the study and showed no significant differences (Table 3). While there were no gains made in reading in the groups receiving the math intervention, this intervention resulted in significant gains on measures of mathematics. Specifically, there were significant main effects of Time Point for the measures of mathematic computational ability (W-J Calculation), timed arithmetic (W-J Math Fluency), and mathematical word problems (W-J Applied Problems), with all measures increasing over this time period. The first two results remain significant after Holm–Bonferroni correction (Table 2), demonstrating that the math intervention was successful and specific in bringing about gains in the domain of math (see Figure 2). There were no significant interactions for Time-Point × Sex during the math intervention.

**TABLE 3:** Pre versus post period of math intervention/null period.

	Mean (SD)			*F* (2,28)	
	Group 1	Group 2	Group 3	*F*-statistic	*p*-Value
***Measures of reading: Woodcock–Johnson***					
Word Identification (single real words)	1.9 (7.1)	−2.6 (6.3)	0.4 (3.5)	1.639	0.212
Word Attack (single pseudowords)	0.4 (5.5)	2.1 (7.3)	−2.8 (4.7)	1.659	0.209
Passage Comprehension (reading comprehension)	0.1 (8.9)	−1.3 (6.7)	2.1 (11.0)	0.374	0.691
***Measures of skills that support reading***					
Lindamood Auditory Conceptualization Test (phonemic awareness)	−0.9 (11.4)	−4.1 (10.0)	−1.4 (5.9)	0.354	0.705
Rapid Naming (naming fluency for letters/numbers)	−1.7 (4.8)	−2.3 (6.6)	1.6 (6.9)	1.075	0.355
Rapid Naming (naming fluency for colors/objects)	−0.4 (8.4)	−4.2 (6.3)	2.8 (9.6)	1.958	0.160
Digit Span (working memory)	2.0 (13.0)	−0.4 (13.0)	−5.0 (10.3)	0.787	0.465
Symbol Imagery (visual imagery/orthographic processing)	−3.7 (11.0)	−3.1 (9.5)	3.9 (6.9)	1.920	0.166

While these analyses allow for direct comparison with previous studies that did not include control periods in their experimental design, we ultimately wanted to test whether gains in reading were statistically greater during the reading intervention period compared to the control periods. For this we conducted a 2 × 2 repeated measures ANOVA for intervention (reading intervention versus math intervention/null period) × measure (change in reading ability on W-J WID versus change in math ability on Calculation standard score) and specifically examined the interaction. The interaction was significant [*F*(1,30) = 18.52, *p* < 0.001], and the *post hoc* test of reading change during the reading intervention versus reading change during the math intervention/null period was significant [*t*(30) = 3.81, *p* = 0.004). During the reading intervention the average reading (W-J WID) standard score change was 6.84, while during the math intervention/null period it was −0.29. Similarly, for the average math (Calculation) standard score change during the math intervention/null period there was an average increase of 7.84, while during the reading intervention scores decreased by −0.26.

Lastly, turning to the follow-up visit, there were no significant differences on the standardized reading measures between the time the children completed the reading intervention and 1 year later (12.4 months on average; assessed in 26 of the original 31 participants; Digit Span was not assessed) indicating that the children maintained the same level of performance they had reached at the end of the intervention. Specifically, as the raw scores on average increased, the standardized measures revealed no significant changes (*p* > 0.05) for any of the eight measures listed above.

### Behavioral Measures as Predictors of Intervention-Induced Gains in Reading

We next investigated whether our behavioral measures of reading and reading-related skills prior to the intervention were predictive of the reading gains made in single real word reading (W-J WID) by the completion of the reading intervention. That is, in the whole group (*n* = 31), each of the eight reading or reading-related measures at Visit 1, along with age, Full IQ, and sex were entered into a multiple regression with change in single real word reading (W-J WID difference before and after the reading intervention) standard score as the dependent variable. The model was not significant [*F*(11,18) = 0.523, *p* = 0.863], and none of the variables contributed to predicting single word reading score change (all *p*-values > 0.05). There was evidence of collinearity among several of the reading and reading-related measures (VIF > 2.5). Removing these specific variables (pseudoword reading, passage comprehension, SI, and age) showed no improvement [*F*(7,22) = 0.788, *p* = 0.605) and again none of the variables contributed to predicting single real word reading score change (all *p*-values > 0.05).

### Brain Activation Changes Following the Reading Intervention

A 2 × 2 ANOVA (Time Point × Sex) to examine changes in brain activity following the reading intervention, and whether sex played a role, yielded no significant results for main effect of Time Point, or for Time Point × Sex interaction (height threshold of *p* < 0.001, uncorrected, extent threshold *p* < 0.05 FWE corrected).

As there were no significant findings in this first analysis examining changes in activation following the reading intervention, there no longer was a need to assess these pre-post differences in comparison to the control periods (math intervention/null).

### Brain Activation as Predictors of Intervention-Induced Gains in Reading

**FIGURE 3:**
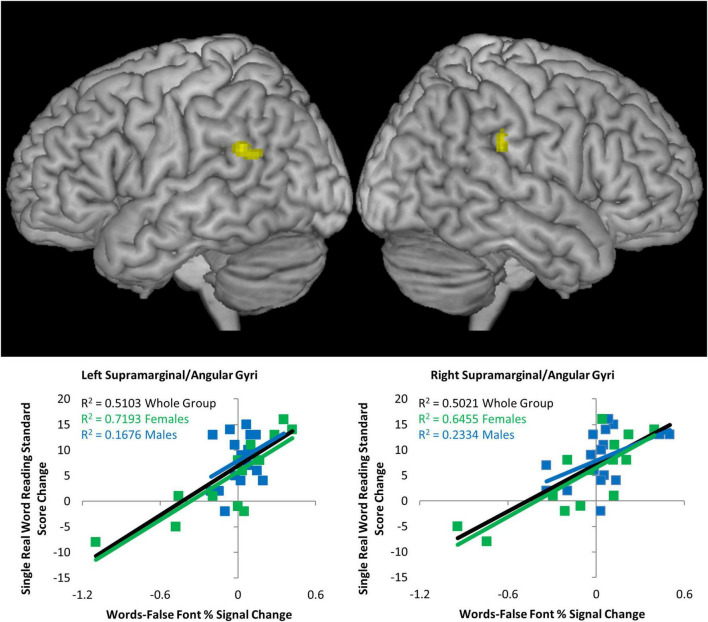
Brain activation predictors of reading gain. Simple regression of Words > False Font activation prior to the intervention versus change in single real word reading (Word Identification) standard score following the reading intervention (*p* < 0.001 uncorrected height threshold, FWE corrected extent threshold *p* < 0.05). **Top:** lateral whole brain views of the whole group relationship between left and right supramarginal/angular gyrus activation and change in score. **Bottom:** signal extracted from each cluster with scatterplots for the whole group (black), females only (green), and males only (blue). *R*^2^ values show strong relationships for both brain regions for females, while in males on the right hemisphere cluster was (barely) significant.

A simple regression analysis of brain activity during reading task at Visit 1 with change in single real word reading (W-J WID difference before and after the reading intervention), revealed two clusters, one in left and the other in right supramarginal/angular gyri (BA 39/40) (height threshold of *p* < 0.001 uncorrected, extent threshold of *p* < 0.05 FWE, corrected) as depicted in Figure 3 (see Table 4). To examine if this predictive relationship was specific to the reading intervention, the signal in these two clusters (activity during reading task at Visit 1) was submitted for correlations with change in single real word reading standard scores during the math intervention (as above, W-J WID standard score differences prior to and immediately after intervention), but neither cluster was significant, showing that the predictive powers in this region were specific to outcomes following the reading intervention.

**TABLE 4:** Coordinates and statistics for regression analysis.

Talairach peak coordinate			Cluster size (voxels)	*T*-statistic	*Z*-score	Anatomical location
*X*	*Y*	*Z*				
Activity during reading at Visit 1 versus change in single real word reading following reading intervention (whole group)						
***Left hemisphere***						
−46	−45	24	205	5.76	4.66	Supramarginal/angular gyri, BA 39/40
***Right hemisphere***						
46	−28	25	126	5.80	4.69	Supramarginal/angular gyri, BA 39/40

This relationship between brain activity during reading task at Time Point 1 and change in reading score was examined for females and males separately. In females only, the relationships were very strong in both the left hemisphere (*r* = 0.848, *p* = 1.3 × 10^–4^) and right hemisphere (*r* = 0.803, *p* = 5.4 × 10^–4^). In males only, the relationships was not significant in the left hemisphere (*r* = 0.409, *p* = 0.103) and barely significant in the right hemisphere (*r* = 0.483, *p* = 0.049) as visualized by scatterplots in Figure 3.

## Discussion

In the present study we studied children with dyslexia: (1) to test for intervention-induced changes in reading performance and in brain activity during reading; and (2) to determine if behavioral measures or brain activity prior to the reading intervention predicted intervention-induced gains in reading. We used a cross-over design allowing us to directly compare reading intervention outcomes with a control period (within-subject control). Overall, the children made strong gains in reading performance (single real word reading, single pseudoword decoding and reading comprehension) as well as the two skills trained during the intervention (phonological and orthographic processing). These gains were specific to the reading intervention as the control math intervention resulted in gains on math but not reading measures, with an ANOVA showing a clear dissociation of the effects of the reading intervention period versus the math/null control period on reading performance versus math performance. However, there were no significant changes in brain activity following the reading intervention. On the other hand, while behavioral measures prior to the onset of the intervention did not predict reading gains made during the reading intervention, brain activation during reading prior to the reading intervention did predict reading gains made during the reading intervention (in left and right supramarginal/angular gyri). Interestingly, while sex was not a significant factor in any of the analyses up until this point, this predictive relationship between pre-intervention brain activity and reading gains following the reading intervention was significant in female subjects, whereas males showed no significant relationship in the left hemisphere and barely in the right hemisphere. These results show that brain activity does not shed light on the neural bases of a successful and enduring reading intervention, but unlike measures of behavior, it identifies regions that signal a level of brain activity that indicates eventual treatment success; and this predictive signal is manifest strongly in females, but not males.

### Behavioral Measures Change Following Reading Intervention

Our study showed performance gains in reading and reading-related skills following the reading intervention. Notably gains occurred on all three measures of reading, namely single real word reading, pseudoword decoding, as well as reading comprehension, the latter ultimately being the raison d’être for reading. No such reading gains occurred in the same children following their control period where some were engaged in a math intervention (active control) with others receiving no intervention at all (null period for developmental control). While one might have expected small carryover effects from the reading intervention into the control periods (due to the within-subject, cross-over design), we did not see gains in reading during the math intervention control period or the null period developmental control. Importantly, an ANOVA confirmed that gains in single word reading performance following the reading intervention was significantly different from any changes in single word reading following the math intervention. Both interventions required the participants’ attention; and they both involved the tutor motivating the child to learn. As such, we can be assured that the reading gains can unequivocally be attributed to the information learned during the reading intervention and were not due to domain-general effects such as attention, or the result of a Hawthorn, or placebo effect. Lastly, these behavioral gains were independent of sex, demonstrating no sex-specific effects on intervention-induced gains in reading.

Overall, our results are similar to those in a prior study of adults with dyslexia (Eden et al., 2004), which used a similar tutoring approach and resulted in measurable gains in single real and pseudoword word reading (but not reading comprehension) as well as in the skills that were trained by the intervention (phonemic awareness and visual imagery). However, children with reading disability in a recent study (Christodoulou et al., 2017; Romeo et al., 2017) did not make gains on these (Word Identification subtest and Word Attack) or other measures of reading, even though the same intervention was used as the one employed here. This underscores the challenges of dyslexia and the fact that not all intervention studies result in a favorable outcome.

Turning to the gains in reading-related measures, it is no surprise that we found gains in those skills trained by the reading intervention, namely, phonemic awareness and visual imagery, demonstrating task-specific training. However, these gains were accompanied by gains in reading, indicating that these improvements in skills that support reading generalized and transferred to reading. Other skills known to support reading acquisition were studied: naming fluency and digit span, which together with phonemic awareness have been described as a set of interrelated phonological processing skills that are impaired in dyslexia due to a core phonological deficit (Wagner and Torgesen, 1987; Stanovich, 1988). As such our results shed light on the fact that these three skills are interrelated yet separate, with gains in one not necessarily accompanied by an equal gain in another. It is also possible that some of these other skills are not as pliable. For example, while some have advocated that working memory can be improved through training (Spencer-Smith and Klingberg, 2015), the strength and generalizability of these gains has been debated (Shipstead et al., 2012; Spencer-Smith and Klingberg, 2015; Nutley and Söderqvist, 2017). However, most likely changes in naming fluency and digit span did not occur because they were not targeted by the intervention. Nevertheless, it is interesting that gains can be made in reading without advancing these two skills.

None of these gains in reading, phonemic awareness or visual imagery were influenced by sex (male versus female). So, while there are behavioral (Wolf and Gow, 1986; Voyer et al., 1995; Weiss et al., 2003; Bornstein et al., 2004) and brain imaging studies (Clements et al., 2006; Sato, 2020; however, see Wallentin, 2009) showing sex-specific effects for language and spatial processing skills, in children with reading disability one sex does not have an advantage over the other when it comes to training the understanding of the sound structure of spoken language and how it maps to print, or the ability to visualize words in one’s mind.

Benefits of the intervention appeared to have longevity, as the standard scores were maintained a year later (as a function of their raw scores increasing), indicating that the students’ progress since the intervention ended was of a magnitude that is consistent with all children in their age group based on this normed testing instrument.

### No Brain Activation Changes Following Reading Intervention

There has been much interest in brain changes following skill acquisition and training. It is known that brain function during object processing in adults who are literate is dramatically different from that of illiterate adults, speaking to the adaptations that occur in the brain as a consequence of learning to read (Dehaene et al., 2010). Successful treatment of dyslexia, it would seem, should be accompanied by changes in brain function. Not only did we observe changes in brain function in adults with dyslexia undergoing a similar intervention (Eden et al., 2004), but prior studies in children have shown increases in activation during letter or word stimuli following reading interventions (Aylward et al., 2003; Temple et al., 2003; Shaywitz et al., 2004). However, as already noted in the Introduction, findings from individual intervention studies are variable. While one meta-analysis of brain imaging studies of reading interventions identified five regions of overlap, specifically in left thalamus, right insula/inferior frontal gyrus, left inferior frontal gyrus, right posterior cingulate, and left middle occipital gyrus, some foci in the meta-analysis were found in only two of the eight studies included. The authors themselves expressed caution in their interpretation of these results because of the variability in the methodologies used in these studies (Barquero et al., 2014). A more recent meta-analysis found no such convergence of results anywhere in the brain (Perdue et al., 2022) and pointed out significant limitations in the existing studies. It is worth noting that ours is the first study to use a within-subject control period to assess activation changes in dyslexia. We also applied a more stringent threshold than previous studies, recognizing that all older studies were accustomed to less stringent practices. Taking all of these factors into consideration our absence of a finding should perhaps not be all that surprising.

However, the question arises whether using a more targeted approach than the whole-brain analysis would have yielded a finding. As such we also conducted a *post hoc* region-of-interest (ROI) analysis. The details of this analysis were not described in section “Materials and Methods” because they followed later, but the approach is similar to that used in other studies (e.g., Brem et al., 2010) and in our prior work (Olulade et al., 2015), and is described in the Supplementary Material with results presented in Supplementary Figure 1. The results yielded no significant changes in specific regions of the fusiform gyrus (home of the visual word form area), even though this very region has shown to be altered by learning to read (Dehaene et al., 2010).

Our interpretation of these results is that there are several possible mechanisms at work, which cannot be differentiated in the current study and could also account for prior variability in the published results. One possibility is that even though gains were made in reading performance, the brain has remained unchanged, such that improved behavior occurs despite persisting functional aberrations. Based on prior, varied findings, we think it is more likely, however, that it has changed, but we are not able to measure these changes because they are too variable to be captured in a group analysis. Such variability would reflect the fact that reading intervention promotes functional changes, but that they occur in different brain regions for different individuals. A likely reason for this would be that if brain regions typically involved in reading do not change following the intervention and instead other brain areas compensate, this compensation may fall to different regions in different individuals. A subset of individuals may be mobilizing traditional reading networks, however, they represent enough of a minority that they are not captured in the group results. In addition to these mechanisms, there will always be some children who did not have brain changes because they did not make significant gains in reading. In this context, however, it is worth noting that a *post hoc* analysis correlating change in reading with change with activity did not yield any findings either. Considering that profiles of dyslexia can be unique, and prior studies on differences in brain anatomy and function in dyslexia have not entirely converged, it is not unreasonable to expect that changes following remediation could show similar variability. In fact, children who have struggled with reading will have received reading instruction or intervention of varying quality, and these prior experiences (which are very difficult to control for) will also be reflected in this variability. It is also possible that any of these sources of variability is reduced in adults, where lower plasticity in adulthood constrains intervention-induced changes to a limited set of brain regions, a possibility that merits further investigations by studying children and adults with dyslexia in the same study. It is also possible that heterogeneity amongst children can be reduced by identifying children at risk for dyslexia (based on a family history of dyslexia) an approach that is helpful in the quest to determine the etiology of dyslexia (Lohvansuu et al., 2021).

### In Females, Brain Activity but Not Behavioral Measures, Predict Gains in Reading Following Intervention

Behavioral measures were unsuccessful at predicting later reading outcome, yet brain activity was. While prior behavioral studies, which demonstrated phonological skills to predict word level reading skills (Hatcher and Hulme, 1999; Torgesen et al., 1999; Catts et al., 2001) set expectations that we would find skills like single real and single pseudoword reading and phonemic awareness to be indicative of later reading gains, we did not. This aligns with a prior report by Hoeft et al. (2011), who found none of 17 reading measures to predict changes in single real word reading over 2.5 years, yet brain activation in right inferior frontal gyrus during a written word rhyming task predicted change in single word reading over the same time period. The current study found that activity in inferior parietal cortex in the left and right hemisphere predicted post-reading intervention gains in single word reading. However, closer examination showed that this effect was driven by the females in the group. Females had strong predictive relationships between activity during reading in the left and right supramarginal/angular gyri and later reading gains, while there was no result for males in the left hemisphere and the effect in the right supramarginal/angular gyrus barely meet significance.

Left temporoparietal cortex represents the indirect route in the dual-route model for reading and is thought to subserve phoneme-grapheme mapping (Pugh et al., 2000, 2001; Coltheart et al., 2001; Jobard et al., 2003). It is thought to be especially important during the early process of learning to read (when new words need to be “sounded out”) and remains engaged into adulthood (Pugh et al., 2001; Turkeltaub et al., 2003; Sandak et al., 2004; Frost et al., 2009). In dyslexia however, this region is underactivated: The left inferior parietal region identified here maps precisely onto the location of less activity in those with dyslexia relative to typical readers identified by meta-analysis (Maisog et al., 2008). Greater engagement of left inferior parietal cortex while processing orthographic and phonological representations of words represent a sign of a brain that is ready to make greater gains in reading once targeted, structured and intense instructions are provided. Of the studies reviewed in the Introduction, one showed left inferior parietal lobule connectivity with middle temporal gyrus during a lexical decision task was predictive of basic reading skill gains following a reading intervention (Aboud et al., 2018). Our results are also consistent with two magnetoencephalography (MEG) studies showing activity in temporoparietal regions (and others) at baseline predicted reading fluency gains following intervention at a 1 year follow-up (Rezaie et al., 2011a,b). Specifically, signal in left and right middle and superior temporal gyri, left supramarginal and angular gyri, left ventral occipitotemporal regions, and right mesial temporal cortex were related to gains in reading fluency.

Why girls but not boys show a relationship where more engagement of left inferior parietal cortex during reading leads to reaping greater benefits from the intervention is not clear. Turning to the literature on the role of sex hormones on brain development, it has been shown that there is a negative correlation between fetal testosterone (*in utero*) and early childhood gray matter volume (8–11 years old) in right TPC, suggesting that the development of this region in males may be modulated by this sex hormone (Lombardo et al., 2012). This in turn may have an impact on brain function, possibly even in the contralateral hemisphere. Post-mortem studies in adults known to have had dyslexia during their lifetime revealed neuronal ectopias (attributed to developmental errors in neuronal migration), primarily in perisylvian regions (Galaburda et al., 1985) and primarily in males (Humphreys et al., 1990). It has been shown that estrogen treatment in women results in increased activation for verbal stimuli, and decreased activation for non-verbal stimuli in the left and right inferior parietal lobule during working memory tasks (Shaywitz et al., 1999). Based on these factors it has been suggested that males and females with dyslexia may have different etiological profiles due to their different hormonal environments (Krafnick and Evans, 2019).

However, it is important to note that the males and females did not differ in the gains they made following the reading intervention. While they may have reached these identical goals in different ways, it is not clear whether there are changes in brain activity following the intervention that are sex-dependent but if there are, we did not capture them. Yet in females, but not males, we were able to identify a left inferior parietal brain region that signals a level or readiness of brain function, promising that the introduction of an intervention will lead to a successful outcome. This suggests some separation of brain function in regions that bring about gains in reading, and regions that signal what may be a certain level of brain function that is required in order to harness the benefits of the intervention, but just in females. Future studies will need to disentangle the relationship between these and directly examine if this predictive relationship is under hormonal influence.

## Conclusion

The results of this study suggest that while it is possible to see significant, specific and enduring gains in reading performance in children/adolescents with dyslexia following intensive treatment, individual variability may explain the fact that we did not observe any change in brain activity following the intervention. On the other hand, brain activity in left TPC predicted reading gains resulting from the intervention, while behavioral measures did not. Interestingly, the predictive powers of brain activity for reading outcome were attributed to the females but not males in our group, suggesting sexual dimorphism in the relationship between brain function during reading and the ability to reap benefits from intensive, structured reading intervention. As a whole, this work suggests there is considerable work to be done to understand brain changes related to reading intervention in order to determine what mechanisms are at work to promote these gains.

## Data Availability Statement

The raw data supporting the conclusions of this article will be made available by the authors, without undue reservation.

## Ethics Statement

The studies involving human participants were reviewed and approved by the Georgetown University (IRB). Written informed consent to participate in this study was provided by the participants’ legal guardian/next of kin.

## Author Contributions

GE and DLF conceived and designed the study. GE and EN were involved in overall study logistics and data collection. AK performed the statistical analyses and together with GE drafted the manuscript. All authors contributed to the manuscript and approved the submission.

## Conflict of Interest

The authors declare that the research was conducted in the absence of any commercial or financial relationships that could be construed as a potential conflict of interest.

## Publisher’s Note

All claims expressed in this article are solely those of the authors and do not necessarily represent those of their affiliated organizations, or those of the publisher, the editors and the reviewers. Any product that may be evaluated in this article, or claim that may be made by its manufacturer, is not guaranteed or endorsed by the publisher.
