# Supplementary material for: The Role of Brain Activity in Characterizing Successful Reading Intervention in Children With Dyslexia
Source: Front Neurosci. 2022 Jun 13;16:898661. doi: 10.3389/fnins.2022.898661 (PMC9234261; doi:10.3389/fnins.2022.898661)
Supplement: Supplementary file 1 [file Data_Sheet_1.docx]

**Supplement: ROI Analysis**

Methods:

A region of interest analysis was used to investigate word specificity within the left OTC visual word form system (VWFS) along a posterior to anterior gradient before and after reading intervention. ROI coordinates are from a previous study from our group Olulade et al. (2015) which investigated VWFS specificity in dyslexic and control children: ROI 1: +/-42, -84, -10; ROI 2: +/-46, -76, -12; ROI 3: +/-46, -68, -14; ROI 4: +/-42, -60, -14; ROI 5: +/-46, -52, -18; and ROI 6: +/- 39, -44, -24. Word-False Font percent signal change from these six spherical ROIs in the left hemisphere was calculated. To directly compare pre- and post-intervention Word-False Font percent signal change in the left hemisphere a repeated measures ANOVA with within subject factors ROI (6 levels) and Time (2 levels), and between subject factor Sex was run.

Results

There was no significant main effect of Time (F(1,29) = 0.0002; p = 0.990) or ROI (F(5,145) = 1.655; p = 0.149), and no significant interactions for ROI x Time (F(5,145) = 1.627; p = 0.156). Looking for effects of sex, there was no significant main effect of Sex (F(1,29) = 2.357; p = 0.136), no ROI x Sex interaction (F(5,145) = 0.815; p = 0.541), no Time x Sex interaction (F(1,29) = 0.159; p = 0.693), and no ROI x Time x Sex interaction (F(5,145) = 0.068; p = 0.997). We also tested if there was a change in the linearity of the effect of ROI following the intervention, however ROI x Time (F(1,29) = 2.264; p = 0.143) and ROI x Time x Sex (F(1,29) = 0.015; p = 0.903) linearity tests were not significant.

**Supplemental Fig 1.**

anterior <------------------- posterior
